# The Application of Active Biomonitoring with the Use of Mosses to Identify Polycyclic Aromatic Hydrocarbons in an Atmospheric Aerosol

**DOI:** 10.3390/molecules26237258

**Published:** 2021-11-30

**Authors:** Paweł Świsłowski, Pavel Hrabák, Stanisław Wacławek, Klára Liskova, Vojtěch Antos, Małgorzata Rajfur, Maria Ząbkowska-Wacławek

**Affiliations:** 1 Institute of Biology, University of Opole, Oleska 22 St., 45-022 Opole, Poland; 2 Institute for Nanomaterials, Advanced Technologies and Innovation, Technical University of Liberec, Studentská St. 1402/2, 461 17 Liberec 1, Czech Republic; pavel.hrabak@tul.cz (P.H.); stanislaw.waclawek@tul.cz (S.W.); klara.liskova1@tul.cz (K.L.); vojtech.antos@tul.cz (V.A.); 3 Institute of Environmental Engineering and Biotechnology, University of Opole, Kominka 6a St., 45-032 Opole, Poland; rajfur@uni.opole.pl; 4 Society of Ecological Chemistry and Engineering, Zawiszaków St. 3/103, 45-288 Opole, Poland; maria.waclawek@o2.pl

**Keywords:** polycyclic aromatic hydrocarbons, bioindicator, moss bag technique

## Abstract

The use of biological indicators of environmental quality is an alternative method of monitoring ecosystem pollution. Various groups of contaminants, including organic ones, can be measured in environmental samples. Polycyclic aromatic hydrocarbons (PAHs) have not yet been determined by the moss bag technique. This technique uses several moss species simultaneously in urban areas to select the best biomonitoring of these compounds, which are dangerous to humans and the environment. In this research, a gas chromatography coupled with mass spectrometry was used for the determination of selected PAHs in three species of mosses: *Pleurozium schreberi, Sphagnum fallax* and *Dicranum polysetum* (active biomonitoring) and for comparison using an air filter reference method for atmospheric aerosol monitoring. The chlorophyll fluorescence of photosystem II (PSII) was also measured to assess changes in moss viability during the study. As a result of the study, the selective accumulation of selected PAHs by mosses was found, with *Pleurozium schreberi* being the best bioindicator—9 out of 13 PAHs compounds were determined in this species. The photosynthetic yield of photosystem (II) decreased by 81% during the exposure time. The relationship between PAHs concentrations in mosses and the total suspended particles (TSP) on the filter indicated the possibility of using this bioindicator to trace PAHs in urban areas and to apply the moss bag technique as a method supporting classical instrumental air monitoring.

## 1. Introduction

Environmental monitoring, including the assessment of exposure to polycyclic aromatic hydrocarbons (PAHs) is based on internal and external exposure [1]. In the first case, we deal with metabolites of PAHs in human body samples [2,3]. The second case is the exposure of people to (working) conditions, where the exposure to PAHs occurs [4,5], which may take the form of both short-term and long-term monitoring [6,7]. PAHs are compounds that have proven mutagenic effects, but they also negatively affect endocrine, reproductive and developmental processes. However, the most serious health effect of human exposure to PAHs is the proven impact of nine compounds from this group on the initiation of the cancer process [8]. These compounds are: anthracene, benz[a]anthracene, benzo[a]pyrene, chrysene, benzo[b]fluoranthene, benzo[k]fluoranthene, dibenz[(a,h]anthracene, benzo[ghi]perylene and indeno[1,2,3-cd]pyrene. There are three routes through which PAHs enter the human body: oral, inhalation and intracutaneous, with the intracutaneous route being considered the least relevant for environmental exposure [9]. Additionally, PAHs concentration can be measured in environmental samples to indicate local long-term atmospheric deposition, identify various PAH sources by fingerprints of individual PAH percentage occurance on the total PAH, or to understand environmental transformation. For this purpose, oxy-, hydroxy- and nitro-PAH derivatives are significant [10].

The natural properties of plants make this group of organisms quite widely used in biological monitoring of PAHs [11]; however, there is a lack of monitoring reports on PAH concentrations in animals in aquatic ecosystems [12,13] or spider webs [14,15]. The use of environmental samples is, therefore, quite common in the monitoring and determination of PAHs environmental fluxes.

The use of mosses as biomonitors of organic pollutants was previously reviewed several times [16,17,18]. This research has a long history, has existed for several decades [19,20] and primarily only concerns the deposition of PAH in different moss species. These studies focus on the use of herbarium specimens as indicators of historical changes and trends in PAH contamination [21,22]. They are used as tools in national and international biomonitoring projects [23,24], and in comparative studies with other bioindicators [25]. One species used in this kind of study, *Pleurozium schreberi* [26,27], is also applied to national surveys [28,29].

Active biomonitoring has an equally long history of PAH monitoring using mosses [30,31,32]. However, the moss bag technique is characterised by the continuous optimisation of the research methodology [33], also used for the determination of PAHs [34,35,36,37]. Similar to passive biomonitoring, studies on PAH determination using the moss bag technique explore the possibility of using different bioindicators simultaneously [38,39,40,41] or long-term studies with an indication of seasonal variations [42]. However, according to the definition of biomonitoring, the study should take into account the physiological state of the bioindicator and consider its vital functions [43]. Meanwhile, active biomonitoring studies using mosses often use devitalized material [38,39]. However, atmospheric concentrations of PAH can be underestimated when dead mosses are used in the moss bag technique [44], and the relevance of using live mosses in research has been demonstrated [45].

PAH pollution in Poland is a large problem in urban agglomerations [46], and their measurement by mosses takes place mainly as passive biomonitoring [47]. There is little work in the literature that integrates active biomonitoring to complement classical instrumental monitoring [48].

The aim of our study was to apply the moss bag technique using three moss species: *Pleurozium schreberi, Sphagnum fallax* and *Dicranum polysetum*, to assess the PAH air pollution of an urban area during the winter period and to demonstrate the selective accumulation properties of these moss species towards PAHs. The study also aimed to demonstrate the applicability of active biomonitoring as a supporting method for the instrumental monitoring of PAHs.

## 2. Results

**Figure 1:**
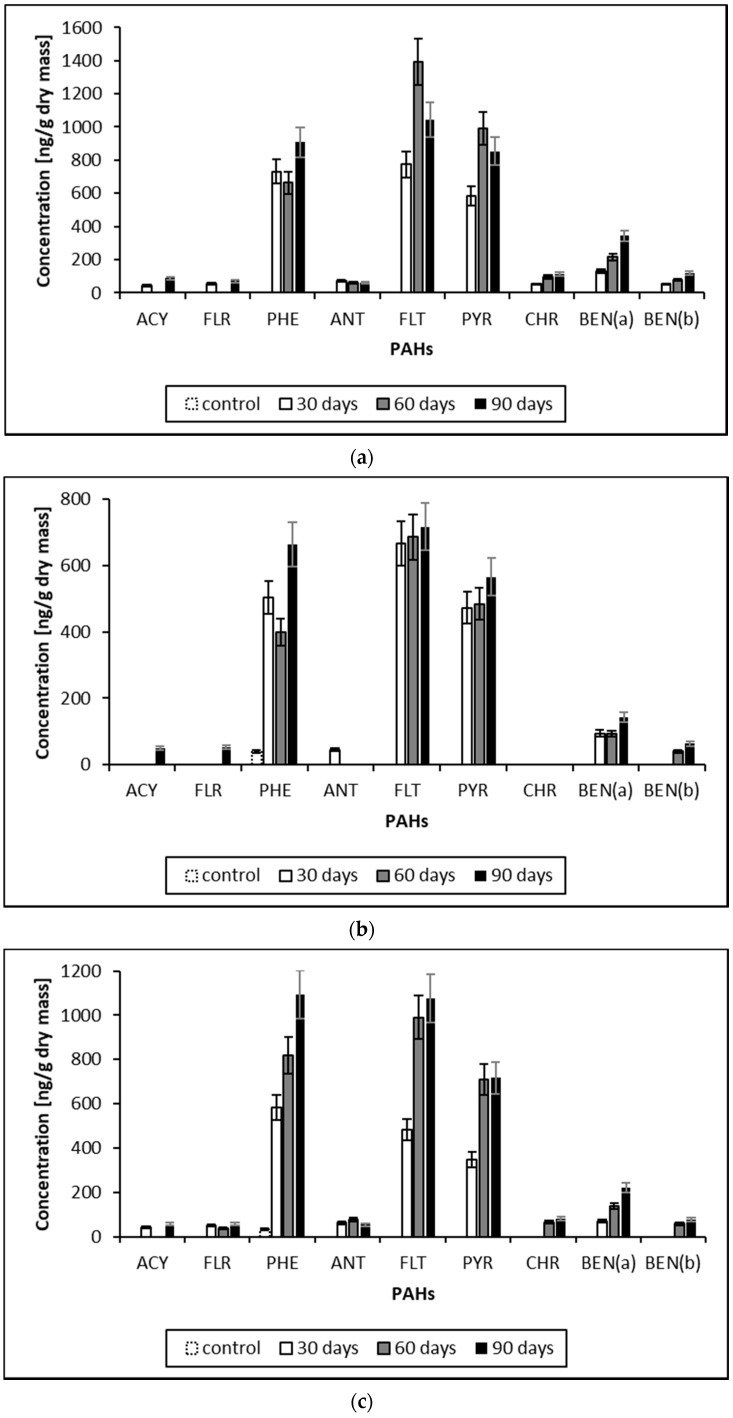
Increments of PAH concentrations accumulated in mosses: (**a**) *Pleurozium schreberi* (Pl); (**b**) *Sphagnum fallax* (Sp); (**c**) *Dicranum polysetum* (Dp). The colors of bars represent growths after the first (white), second (grey) and third (black) months of exposure. The white bar outlined with a dashed line represents the concentration of the control (moss sample that has not been exposed). The absence of a bar indicates that the concentration in the control sample was <10.0 ng/g limit of detection (LOD). PAH concentration increments, i.e., the concentration measured in the post-exposure sample (Cm) subtracted from the concentration in the control sample (Cc): Cm—Cc. Abbreviations for PAHs: Acenaphthylene (ACY); Fluorene (FLR); Phenanthrene (PHE); Anthracene (ANT); Fluoranthene (FLT); Pyrene (PYR); Chrysene (CHR); Benzo(a)anthracene (BEN(a)); Ben-zo(b)fluoranthene (BEN(b)).

Figure 1 shows the concentrations of selected PAHs accumulated by three moss species during a 12-week exposure.

The concentrations of PAHs accumulated by mosses, shown in Figure 1, indicate a monotonic accumulation with the duration of the exposure period for most compounds. For all three species, this is eight compounds (after the second and third month of exposure); for Pl and Dp, it is also CHR. Four PAH compounds have the highest concentrations irrespective of exposure duration; these are PHE, FLT, PYR and BEN(a). Based on the statistical significance between PAH concentrations accumulated in the three moss species calculated by the Wilcoxon test, p was: Pl and Sp < 0.001 (0.0003), Pl and Dp < 0.05 (0.011) and Sp and Dp < 0.05 (0.022). Therefore, the species can be ranked with respect to the value of PAH concentrations and irrespective of time according to the co-efficient: Pl > Dp > Sp.

The measured value of actual photochemical efficiency (yield) indicates a decrease in value during moss exposure in winter. The mean initial value in the control samples was 0.696, and after three months, it was 0.133. The decrease in photosynthetic activity by 81.0% after 12 weeks of exposure was caused by environmental stress due to changeable weather conditions and air pollution (due to the heating season), including PAHs and heavy metals (work in progress). In the next step, the concentrations of PAHs deposited in TSP and accumulated on the filter were analyzed by instrumental monitoring (Table 1)

**Figure 2:**
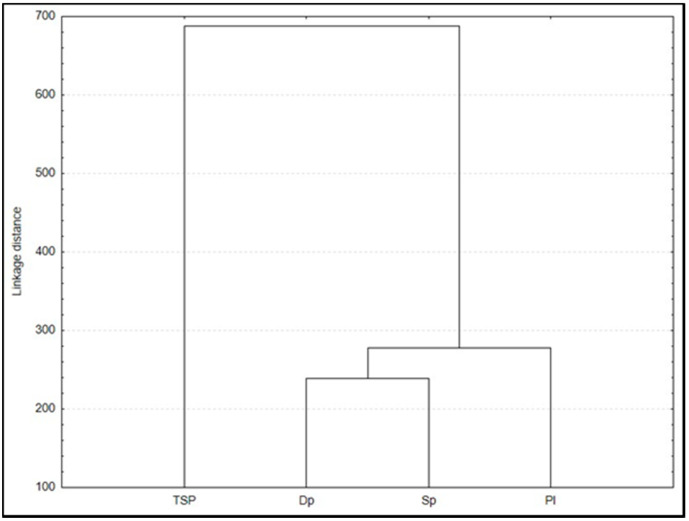
Cluster analysis of PAHs concentrations in three moss species and in TSP on the filter (Single linkage, Euclidean distance).

The values presented in Table 1 indicate high daily increments of extracted PAHs on the filter. In relation to mosses, four additional compounds were also determined in the TSP on the filter: BEN(k), BEN(a)PYR, IND and DIB. The values are disproportionately high compared to the concentrations accumulated by mosses. In the TSP, 11 PAH compounds were determined on the filter. The common compounds determined in all moss species and on the filter were: PHE, ANT, FLT, PYR and BEN(a). Figure 2 shows the correlation of the concentrations of the compounds mentioned above accumulated by the mosses (Pl, Sp, Dp) in relation to the same PAH compounds deposited on the filter (TSP).

Cluster analysis between PAHs concentration on filter and mosses reveals clusters with varying linkage distance. The results obtained by mosses are significantly different from the ones obtained by classical monitoring. The results indicate a weak relationship between the concentrations of PAHs in the moss bag active biomonitoring technique and classical instrumental monitoring, which shows the differing performances of the two techniques.

## 3. Discussion

To date, there has been little work in active biomonitoring where the species used were also involved in the biological monitoring of post-airborne PAH contamination. Two papers concern the application of the moss bag technique for the species *Pleurozium schreberi* [31,49]. In the first paper, where PAH concentrations in mosses are compared to PAH contamination in snow samples, the determined compounds differ from those determined in our study. *P. schreberi* in the presented study also accumulated na-phtalene, acenapthene, benzo(a)pyrene, indenopyrene, dibenzo(ah)anthracene and ben-zo(ghi)perylene. However, in our study, the concentrations determined for common PAH compounds were higher after the same exposure time. The determination of additional PAHs may have been influenced by the location of the study—a motorway, and thus the impact of car traffic, exhaust emissions. In addition, the authors found an advantage of using the moss bag technique in PAH deposition over snow sampling for this type of study [31]. In the second case, where the same species (Ps) were also exposed, moss transplantation using the moss bag technique lasted only one week in the cemeteries during the All Saints’ Day period. The study indicated that the burning of candles caused the emission of selected PAHs [49]: benzo[b]fluoranthene and benzo[k]fluoranthene, dibenzo[a,h]anthracene, naphthalene, pyrene, indeno[1,2,3-cd]pyrene and ben-zo[g,h,i]perylene. Both the concentrations obtained and the PAH compounds determined were influenced by the samples’ times and places of exposure. Two further national studies also investigated the use of *P. schreberi* in PAH monitoring, but this time by the box moss transplantation technique. The analyses showed that the determined PAH compounds were similar to those determined in this study. As in previous studies, the concentrations were very low, despite the longer exposure time (6 months) [50]. The low concentrations obtained may be due to the exposure method used [50,51]. 

Due to the lack of studies in the literature analyzing the PAH content of the other two species used by us (Sp and Dp), we decided to present selected literature data in tabular form on the application of the moss bag technique using other moss species for PAH determination (Table 2). 

The examples from the literature, presented in Table 2, show the variation in the accumulation of PAHs by mosses at different times, places and during different exposure times. Our study presents PAHs increments after three months of exposure that ended in January, during the heating season. The significant increases in PAHs concentrations for the three moss species were influenced by the season of the study, as well as by the poor air quality in the area, which was previously reported [56,57,58]. Therefore, the time of study should be taken into account, as mosses show seasonality/periodicity of increased contamination depending on the study period [55,59]. Of the data presented, *P. schreberi*, which is also a passive bioindicator, demonstrated the highest concentration of adsorbed PAHs in our study [26,60]. The results of actual photochemical efficiency (yield) indicate that live mosses, when exposed to adverse conditions (environmental stress [61]), spend most of their exposure time in a physiological state (cryptobiosis) in which vital parameters are very low, but compounds are accumulated. Thus, it is not advisable to exclude measurements of viability during biomonitoring studies [39,43]. The influence of environmental factors has a significant impact on the life of plants (including mosses) [62] and the ways in which PAHs accumulate in a plant are also dependent on environmental conditions and the characteristics of the plant itself (how it is prepared for research) [16,59,63].

It is essential for biomonitoring to be integrated with classical monitoring in order to demonstrate its applicability as a complementary method to routine instrumental measurements [48]. In one study, the concentrations of PAHs in mosses and active accumulators were correlated where the coefficient r of Pearson showed a significant correlation R = 0.67 [53]. It is not possible to replace instrumental monitoring with biomonitoring (see Figure 2), but the presented results indicate the possibility of using the moss bag technique as a method of supporting the classical instrumental monitoring of airborne PAHs in urban areas.

In food and environmental matrices, Benzo[a]pyrene (BEN(a)PYR) is probably the most studied PAH. However, in many cases, BEN(a)PYR represents only 1–20% of the total PAH concentration [61]. In 2002, the European Commission’s Scientific Committee on Food (SCF) recommended that, in addition to BEN(a)PYR, other compounds in the PAH group should also be considered for the determination of carcinogenic effects [64]. The air pollution indicator for PAHs is the concentration of benzo(a)pyrene, which, according to Directive 2004/107/EC of the European Parliament and of the Council of 15 December 2004 (related to arsenic, cadmium, mercury, nickel and polycyclic aromatic hydrocarbons in ambient air) should not exceed 1 ng/m^3^ [65]. In mosses, the concentration of BEN(a)PYR was below the limit of quantification of the analytical method used, but it should be remembered that living mosses will only accumulate bioavailable forms of analytes. At the same time, high concentrations of other mutagenic PAHs were found both in TSP and in the living biomonitor (in samples of three moss species). Analyzing the data from the work of Sapota [66] and comparing them with the test results obtained herein, PAHs with high relative carcinogenicity and mutagenicity were determined in mosses, e.g., 0.62 for benzo(a)anthracene and 0.37 for chrysene.

## 4. Materials and Methods

The species used for this study were the mosses *P. schreberi, S. fallax* and *D. polysetum*, which are used in biomonitoring studies [67,68,69], including PAH contamination [51]. They were collected in October 2020 from forests in the Swietokrzyskie Voivodship in southeastern Poland, which can be considered a clean background site. Mosses were collected at least 5 m away from the canopy of the trees, so as to not be directly exposed to precipitation (only the green parts of mosses were taken) [70].

Moss samples were taken and prepared before exposure according to a pre-developed methodology [71]. Next, 2 g of mosses were each packed into nylon nets and exposed in bags. The control sample was not exposed and was left for pre-exposure analyses of PAHs concentrations to assess background pollution. Moss samples were suspended on the viewing terrace of one Opole University buildings (Opole, Poland). The mosses were exposed for 3 months during the winter season (27 October 2020–27 January 2021). Nine bags were removed for each month of exposure (3 bags for each species). At the same time, total suspended particles were collected on QM-A quartz filters (Whatman, 47 mm diameter). The sampling time was 24 h. Airflow in PNS3D15/LVS3D dust collector was measured as 2.3 m^3^/h following the standard procedure [72].

One gram of each sample was extracted with 10 mL of methanol (HPLC grade) using lab shaker GFL 3006. All samples were extracted for 24 h. The eluate obtained was subjected to gas chromatographic analysis, which was carried out using a Thermo Trace 1310 a gas chromatograph equipped with a triple quadrupole MS detector Thermo TSQTM 8000 Evo, an autosampler CTC Analytics AG, PAL LHX-xt and a programmed temperature vaporizing injector. Analytical column SCION 5 ms (30 m × 0.25 mm × 0.25 µm) was used. The temperature programme of the chromatographic oven started at 70 °C, graduated, at first, by 15 °C per min to 200 °C and was maintained for 5 min, followed by a temperature gradient of 8 °C per min to final temperature of 320 °C, which was maintained for 5 min. The carrier gas (helium 5.0) flow was adjusted to 2 mL per min. 

Calibration with internal standard was performed for the determination of all PAH. Four deuterated standards: naphthalene—d8, phenanthrene—d10, chrysene—d12 and perylene—d12, and mixed-standard Dr. Ehrenstorfer PAH-Mix 9, were used for determination of PAH. Limits of quantification were 1 µg·mL^−1^ for each of the analytes. Regarding the analytical methods, a detailed description of individual PAHs MS parameters is provided in the Supplementary Material.

Chlorophyll fluorescence of photosystem II, actual photochemical efficiency (yield) was measured using the modulated portable fluorometer (Opti-Sciences, Hudson, NH, USA) under ambient light conditions [73].

The Lilliefors modifications of the Kolmogorov–Smirnov test failed to prove a hypothesis about data normality [74]. Therefore, differences between moss species, in terms of the concentration of PAHs that they contained, were evaluated by the Wilcoxon test [75].

## 5. Conclusions

The results of the biomonitoring study demonstrate the applicability of the moss bag technique to determine selected PAHs. In the active biomonitoring of air pollution by these compounds, the so-far rarely used species, *P. schreberi,* and two others, *S. fallax* and *D. polysetum*, were chosen. The increments in PAH concentrations in the mosses indicate that the former proved to be the best biomonitoring species. This species was also most suitable from the perspective of areal abundance, which qualified it for the mapping purposes. Biological monitoring showed an accumulation of PAHs at a specific time, e.g., winter and heating seasons, which is confirmed by accumulated concentrations by mosses, as well as those deposited in TSP on the filter, where an additional 4 PAHs compounds were found. The viability of the mosses decreased to low values during the exposure, which shows the state of moss cryptobiosis as a physiological state serving their survival in unfavorable environmental conditions, which included air pollution with PAHs. The correlation between PAH in mosses and in TSP on the filter indicates that the use of environmental samples can support classical monitoring in urban areas. Further research should focus on the optimization and standardization of the moss bag technique in PAH monitoring.

## Figures and Tables

**Table 1:** Mean concentrations of PAHs accumulated on air filter.

Acronym	C_PAH_ [µg/g]
PHE	28.5
ANT	6.28
FLT	143
PYR	137
CHR	123
BEN(a)	138
BEN(b)	144
BEN(k)	41.6
BEN(a)PYR	91.3
IND	73.9
DIB	15.0

PAH abbreviations: Benzo(k)fluoranthene (BEN(k)); Benzo(a)pyrene (BEN(a)PYR); In-deno(1.2.3)-cd_pyrene (IND); Dibenzo(a.h)anthracene (DIB).

**Table 2:** Comparison of PAH concentrations accumulated by other moss species in comparison to this work.

	Mean of PAHs Concentration [ng/g]										
Species	ACY	FLR	PHE	ANT	FLT	PYR	CHR	BEN(a)	BEN(b)	Time of Exposure	References *
*P. schreberi*	88.0	69.5	908	60.2	1043	853	111	343	116	12 weeks	This study
*S. fallax*	49.4	53.1	665	<10.0	717	566	<10.0	144	62.9	12 weeks	This study
*D. polysetum*	57.1	57.1	1093	53.0	1077	718	81.6	220	77.5	12 weeks	This study
*H. splendens*	23	24	413	59	708	835	313	188	290	4 weeks	[52]
*H. cupressiforme*	n.d.	n.d.	84	14	273	327	27	50	18	3 weeks	[53]
*H. cupressiforme*	n.d.	n.d.	14.7	2.11	31.5	43.3	13.6	19.7	n.d.	4 weeks	[41]
*H. cupressiforme*	n.d.	2.28	35.4	<DL	29.9	17.6	11.6	1.19	5.07	6 weeks	[38]
*H.cupressiforme*	−1.7 **	−2.9 **	16	0.3	25.6	29.9	11.7	3.7	2.8	6 weeks	[43]
*H.cupressiforme*	10	26	367	9.34	309	232	57	12	17	6 weeks	[39]
*P. purum*	n.d.	15.1	60.6	6.06	57.6	208	14.5	5.30	n.d	8 weeks	[54]
*S. girgensohnii*	10.4	14.6	90.4	10.2	206	175.9	181.5	42.3	92.4	8 weeks	[55]

* When more than one study site appeared in a given publication shown in Table 2, the study site where PAHs content in mosses was highest, or where their labeled amount was highest, was selected for comparison; n.d.—no data; <DL and <10.0—below the detection limit. ** Post-minus, pre-exposure difference is shown; therefore, negative numbers are possible.

## Data Availability

The datasets used and/or analyzed during the current study are available from the corresponding author on reasonable request.

## Supplementary Materials

The following are available online, Table S1: MS parameters of PAH determination method.

## Sample Availability

Samples of the compounds are available from the authors.
