# Supplementary material for: The Application of Active Biomonitoring with the Use of Mosses to Identify Polycyclic Aromatic Hydrocarbons in an Atmospheric Aerosol
Source: Molecules. 2021 Nov 30;26(23):7258. doi: 10.3390/molecules26237258 (PMC8659324; doi:10.3390/molecules26237258)

Supplementary Information

Table S1. MS parameters of PAH determination method

**Analytes**	**Retention time min**	**Parent ion m/z**	**Product ion m/z**	**Collision energy eV**	**ISTD**	**LoD ng·g^{-1}**	**LoQ ng·g^{-1}**
Naphthalene	4.45	128	102	20	1	0.29	0.96
Acenaphthylene	7.02	152	126	20	1	0.26	0.88
Acenaphthene	7.29	153	151	40	1	0.29	0.96
Fluorene	8.11	165	163	30	2	0.38	1.28
Phenanthrene	9.82	178	152	25	2	0.58	1.92
Anthracene	9.93	178	176	20	2	0.62	2.08
Fluoranthene	13.96	202	200	30	2	0.67	2.24
Pyrene	14.98	202	200	30	3	0.55	1.84
Chrysene	19.87	202	200	35	3	0.50	1.68
Benz(*a)*anthracene	19.98	228	226	30	3	0.62	2.08
Benzo(*b)*fluoranthene	23.27	252	250	30	4	0.58	1.92
Benzo(k)fluoranthene	23.35	252	250	30	4	0.65	2.16
Benzo[*a*]pyrene	24.14	252	250	30	4	0.53	1.76
Indeno(*1.2.3-cd)*pyrene	26.86	276	274	40	4	0.41	1.36
Dibenzo(*a.h)*anthracene	26.95	278	276	30	4	0.46	1.52
Benzo[*ghi*]perylene	27.41	276	274	30	4	0.30	0.99
**Internal standards**							
ISTD Naphthalene D8	4.45	136	108	25	1		
ISTD Phenanthrene D10	9.81	188	160	30	2		
ISTD Chrysene D12	19.90	240	236	30	3		
ISTD Perylene D12	24.31	264	260	30	4		

Linear calibration range for each analytes was from 1,25 ng·ml^{-1} – 250 ng·ml^{-1}.

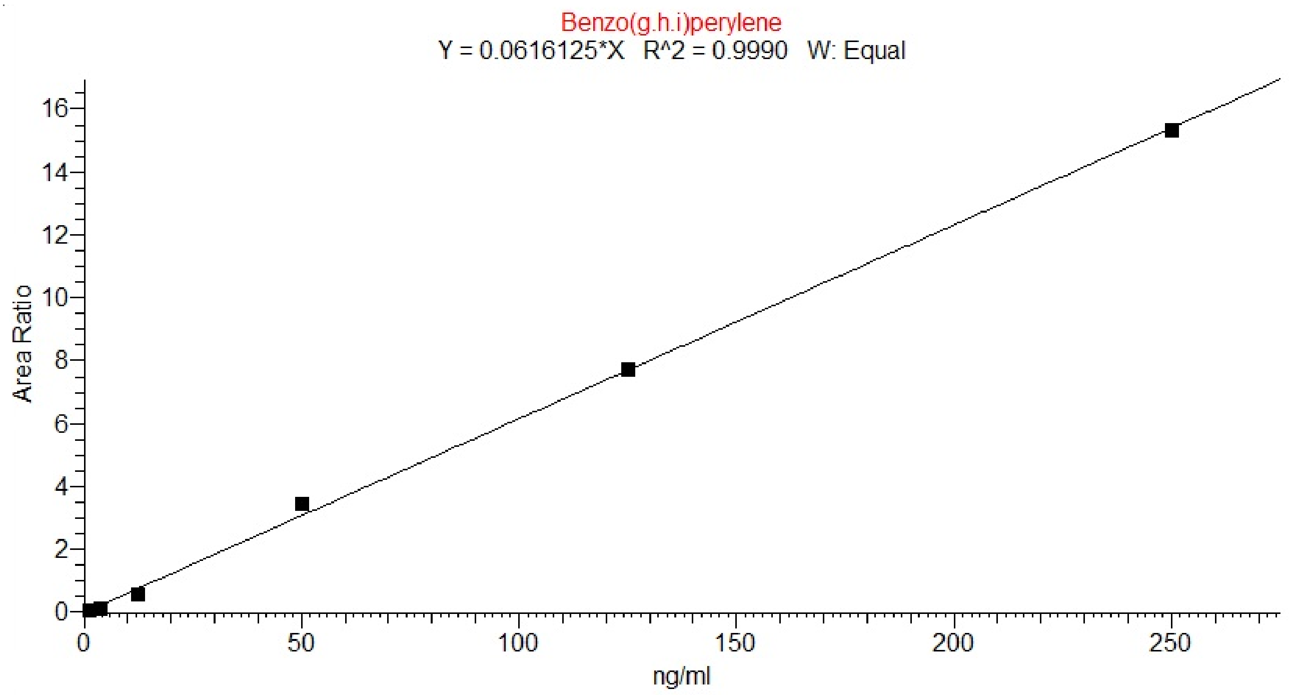

Supplement: Supplementary file 1 [file molecules-26-07258-s001.zip › molecules-1434203-supplementary.pdf]
